# Uræmic Convulsions Treated by Pilocarpine

**Published:** 1888-03

**Authors:** 


### A CASE OF URÆMIC CONVULSIONS SUCCESSFULLY TREATED BY PILOCARPINE.

Dr. W. J. F. Churchouse in the *British Medical Journal* says: J. M., aged 21, fell ill with scarlet fever on October 13. He went on well until November 3, when symptoms of nephritis showed themselves; the attack was slight, and appeared to improve rapidly under treatment.

On November 10 I found him in uræmic convulsions ; the fits occurred every few minutes. My friend Mr. Cox saw him with me, and with his concurrence, I injected one-third of a grain of pilocarpine into the front of the forearm ; the patient soon began to sweat profusely, and the convulsions gradually diminished both in force and frequency ; they recurred in the early part of the night about every fifteen or twenty minutes, but with longer intervals towards morning ; at 8.30 a. m. I repeated the pilocarpine, and the intervals between the convulsions became still longer ; in the afternoon, when he became sensible enough to swallow, I gave him one-third of a grain of elaterium ; this acted freely, and he probably passed a little urine at the same time, otherwise he had passed none since 7 or 8 the previous evening ; as the convulsions still recurred at frequent though longer intervals, I again injected one-third of a grain of pilocarpine at 9.30 p. m. During the night he had a few slight fits, and in the morning about 11 a. m., I gave him another injection of pilocarpine, and the last fit occurred about 4 p. m. on that day. In the evening I again gave him one-third of a grain of elaterium, which acted freely, and he now passed more urine, the urine having been very scant up to this time.

On November 15 he had symptoms of more convulsions coming on, but I gave him another injection of pilocarpine, and this passed off.

He lay in a semi-comatose condition from November 12 to 17, swallowing what was put in his mouth, but totally unconscious of everything going on around him, and taking no notice of anything ; on November 17 he was maniacal, and on November 20 he could not be kept in bed. During the whole of this time the bowels had been kept open by occasional doses of elaterium, and he had taken a mixture containing liq. ferri perchlor. and liq. ammon. acetas. On November 22 he became sensible and talked rationally, but had no recollection of anything that had occurred since November 10 ; on November 25, for the first time since the commencement of his illness, there was no trace of albumen, and he improved rapidly ; on November 28 he got up and dressed, and from that time made a good recovery, and is now again following his trade as a shoemaker.

I think we have in pilocarpine a most prompt and valuable diaphoretic.

